# The Moderating Effect of Coping on Stress and Childhood Obesity-Related Health Behaviors among Non-Hispanic Black Caregivers

**DOI:** 10.1007/s40615-025-02408-7

**Published:** 2025-04-02

**Authors:** Elizabeth Prout Parks, Shiriki Kumanyika, Reneé H. Moore, E. Thomaseo Burton, Timothy Khalil, Lisa M. Lewis, Frances K. Barg, Andrew C. Pool, Yasmeen Bruton, David B. Sarwer, Anne E. Kazak

**Affiliations:** 1 Health & Wellbeing Program, Children’s Hospital of Philadelphia, Philadelphia, PA USA; 2 Division of Gastroenterology, Hepatology, and Nutrition, Children’s Hospital of Philadelphia, Philadelphia, PA USA; 3 Perelman School of Medicine, University of Pennsylvania, Philadelphia, PA USA; 4 Dornsife School of Public Health, Drexel University, Philadelphia, PA USA; 5 Biostatistics Collaboration Center, Drexel University, Philadelphia, PA USA; 6 Department of Child and Adolescent Psychiatry and Behavioral Sciences, Children’s Hospital of Philadelphia, Philadelphia, PA 19104 USA; 7 School of Nursing, University of Pennsylvania, Philadelphia, PA USA; 8 Department of Family Medicine and Community Health, University of Pennsylvania, Philadelphia, PA USA; 9 Department of Anthropology, University of Pennsylvania, Philadelphia, PA USA; 10 Center for Parent and Teen Communication, Craig-Dalsimer Division of Adolescent Medicine, Children’s Hospital of Philadelphia, Philadelphia, PA USA; 11 Center for Obesity Research and Education, College of Public Health, Temple University, Philadelphia, PA USA; 12 Nemours Children’s Health, Wilmington, DE USA; 13 Department of Pediatrics, Sidney Kimmel School of Medicine, Thomas Jefferson University, Philadelphia, PA USA

**Keywords:** Parent stress, Race-related stress, Religious coping, Weight status, Child health

## Abstract

Child health behaviors and weight status may be affected by caregivers’ perception and experience of stress. However, little is known about the influence of caregiver coping strategies on childhood overweight and obesity, particularly among non-Hispanic Black caregivers. This study examined associations among specific caregiver stress types (i.e., general, parenting, race-related), child weight status, and health-related behaviors (i.e., intake of fruits and vegetables, consumption of fast food, engagement in physical activity) as well as the moderating effect of caregiver coping strategies. In addition to general coping, the study examined the role of religious coping. This cross-sectional study included 157 non-Hispanic Black caregiver and child (aged 3–7 years) dyads; all caregivers identified as Christian Protestant. Logistic regression models were fit to explore the associations among caregiver stress and child outcomes and to investigate moderation effects of caregiver coping. Models were adjusted for sociodemographic covariates. Association between parenting stress and child weight status was significantly moderated by acceptance coping while the relationship between general stress and child sugary drink intake was moderated by emotional coping. The combination of three stress types was significantly associated with increased child fast food intake. Future research should examine specific coping strategies to address varying levels and types of stress experienced by caregivers from minoritized backgrounds.

## Introduction

Childhood overweight and obesity are significant clinical and public health issues that affect more than one-third of youth in the United States [1]. Non-Hispanic Black children are at higher risk for the development of overweight, obesity, and related co-morbidities (e.g., type 2 diabetes, hypertension, depressive symptoms) compared to non-Hispanic White children, which highlights the importance of considering cultural and contextual factors in prevention and intervention efforts [2, 3].

Prevention and intervention of childhood overweight and obesity tend to focus on familial lifestyle behaviors [1]. In particular, parental caregivers play a key role in children’s development of health behaviors through modeling as well as facilitating or limiting access to healthy choices, such as meeting daily dietary and activity recommendations [4–6]. Caregivers’ influence on child health behaviors and weight status may be affected by many factors, including caregiver stress [7–9]. For example, researchers have reported associations between higher levels of caregiver stress and less availability of fruits, vegetables, and healthy drinks in the home as well as limited opportunities for children to meet physical activity guidelines [10, 11]. An increased focus on the role of stress in non-Hispanic Black caregivers may provide important insights for targeted and equitable prevention and intervention.

Caregivers are susceptible to multiple types of cumulative stress, and racial/ethnic minoritized caregivers tend to be more affected by stress than majority group counterparts; this stress may influence health-related parenting practices [7, 8, 12–15]. Of note, non-Hispanic Black caregivers may experience additional stress related to perceived and experienced racism, which is known to be associated with increased risk of obesity, diabetes, and cardiovascular disease in adults which could, in turn, increase child risk [11, 16–18]. To date, no studies have examined the influence of caregiver perceived racism, alone or in the presence of other stress types, on childhood weight status or related health behaviors.

While caregiver use of coping to manage stress has been shown to influence child health outcomes [19, 20], the moderating role of caregiver coping on caregiver stress and childhood obesity-related behaviors (e.g., intake of fruits and vegetables, consumption of fast food, engagement in physical activity) remains understudied, particularly among non-Hispanic Black caregivers. Moreover, examining the use of spiritual and religious coping strategies (e.g., prayer, attendance at religious services, meditation, reading scripture), which have been shown to be beneficial in non-Hispanic Black individuals’ health behaviors and health outcomes [21–23], may provide greater insight into the development of meaningful intervention strategies.

**Fig. 1:**
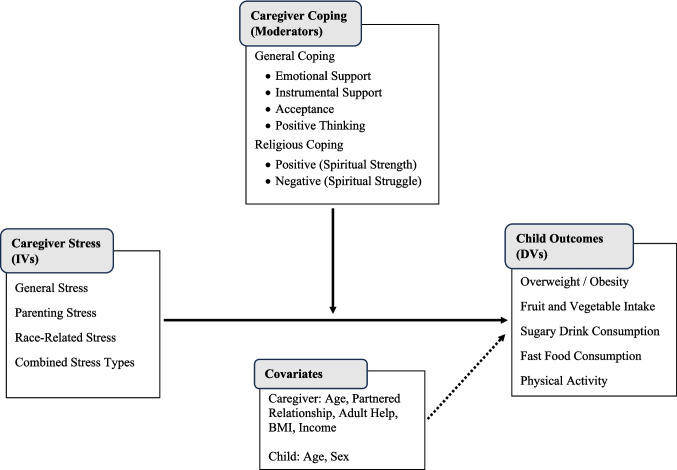
Conceptual Model. *Note*. IVs = independent variables; DVs = dependent variables; BMI = body mass index

Considering the dearth of empirical knowledge on the influence of caregiver coping styles towards stress and its effect on childhood overweight and obesity and related behaviors, the present study aimed to investigate associations between caregiver stress types and child weight status, including weight-related health behaviors. An additional aim of this study was to examine the moderating effect of caregiver coping strategies on the hypothesized relationship between caregiver stress and child overweight or obesity, intake of fruits and vegetables, consumption of sugary drinks, consumption of fast food, and engagement in physical activity (see Fig. 1).

## Methods

### Participants and Procedures

Non-Hispanic Black participants for this cross-sectional study were recruited from the metropolitan Philadelphia area between January 2015 and October 2016 using a variety of strategies: radio announcements, newspaper advertisements, email flyers to patients of a local pediatric healthcare network, and announcements and recruiting events at four local churches with primarily non-Hispanic Black memberships. Potential participants were screened by telephone. Inclusion criteria included being the primary adult caregiver (i.e., parent, grandparent, legal guardian) of a child ages 3–7 years. Because an overall study goal was assessment of the role of religion and spirituality in coping with stress, Protestant Christian faith and church attendance were additional inclusion criteria. In the case that more than one child in the household was between the ages of 3 and 7 years, the child whose last birthday was closest to the date of enrollment was identified as the target child for the study [24].

To distinguish between psychological distress and stress, potential participants were administered the MINI-International Neuropsychological Interview [25] and were excluded if they reported having psychosis, bipolar disorder, schizophrenia, or untreated depression or anxiety. Child participants were excluded if caregivers reported any of the aforementioned mental health diagnoses in their child or if their child had genomic or syndromic obesity, developmental delays, or any medical conditions or prescribed medications that influenced weight gain.

Data collection visits were scheduled for caregiver / child dyads at participants’ church or at a centralized research site; data were collected by trained research assistants. This approximately 1.5-h visit entailed caregiver and child height and weight measurements, survey administration, and orientation to use of accelerometer devices. Following the study visit, caregivers were contacted by a dietitian via telephone and asked to complete three 24-h dietary recalls (two weekdays, one weekend day) of their own and their child’s food and beverage intake. The study was approved by the Children’s Hospital of Philadelphia Institutional Review Board. Parent informed consent and child assent in children aged 7 years were obtained. Data supporting the findings of this study are available upon reasonable request.

### Measures

#### Sociodemographic Variables

A brief questionnaire was used to obtain sociodemographic information including caregiver and child age, sex, race/ethnicity, church membership, educational attainment, employment status, and family income.

#### Anthropometric Measurements

Caregiver and child height (to the nearest 0.1 cm) and weight (to the nearest 0.1 kg) were used to calculate body mass index (BMI; kg/m^2^) and were measured by study staff using a digital electronic scale (Seca, Munich, Germany) and stadiometer (Holtain, Crymych, UK), respectively. Participants removed shoes and excess clothing (e.g., coats, sweaters) and measurements were taken in triplicate and averaged. BMI for age and sex and BMI-z scores were calculated. BMI percentile cut-offs were used to classify participants into overweight or obesity categories [26].

#### Caregiver Stress

*General stress.* The Perceived Stress Scale (PSS-10) [27] is a 10-item self-report measure of general stress. Items assess an individual’s perception of their ability over the past month to control important aspects of life, confidence to handle personal problems, and ability to overcome difficulties. Responses to each item are rated on a 5-point Likert-type scale, from 0 (*never*) to 4 (*very often*) and summed to create a total PSS score, with higher scores indicating more stress.

*Parenting stress.* The Parenting Stress Index – Short Form (PSI-SF) [28] is a 36-item caregiver-report measure of stress associated with parenting a child ≤ 12 years of age. The questionnaire comprises three subscales rated on a 5-point Likert scale (*strongly disagree* to *strongly agree*). Subscale scores represent the sum of each respective domain, with higher scores indicating greater endorsement of the domain: *Parental Distress*, *Parent–Child Dysfunctional Interactions*, and *Difficult Child*.

*Race-related stress.* The Prolonged Activation and Anticipatory Race-Related Stress Scale (PARS) [29] is a 17-item self-report measure of anticipatory physiological and psychological racism-related stress. The questionnaire is made up of four subscales (i.e., Perseverative Cognition, Anticipatory Race-Related Stress-Physiological, Anticipatory Race-Related Stress-Psychological, and Secondary Appraisal), with responses rated on a 7-point Likert-type scale (*strongly disagree* to *strongly agree*).

*Combined stress types*. A composite of stress types was developed by counting the number of stress measures (i.e., general, parenting, and race-related) with high stress scores, resulting in an overall stress score of 0, 1, 2, or 3. High stress for the individual stress measures was determined as follows: a parenting stress score ≥ 85th percentile, and perceived and race-related stress scores in the upper tertile.

#### Caregiver Coping

*General coping*. The Coping Orientation to Problems Experienced Inventory (Brief-COPE) [30] is a 28-item self-report measure of adaptive and maladaptive strategies used to cope with stressful life events. The measure comprises three overarching coping styles (i.e., problem-focused, emotion-focused, and avoidant), with responses rated on a 4-point Likert-type scale from 1 (*I usually do not do this at all*) to 4 (*I usually do this a lot*). The Brief-COPE can be further broken down to facets (e.g., emotional support, acceptance, religion) that can be used to pinpoint specific coping strategies.

*Religious coping.* The Brief Religious Coping Scale (Brief RCOPE) [31] is a 14-item self-report measure that examines the use of religion and spirituality to cope with life stressors. The measure has two subscales, positive and negative, which are scored on a 4-point Likert-type scale from 0 (*not at all*) to 4 (*a great deal*).

#### Child Health Behaviors

*Dietary recall*. Trained registered dietitians contacted caregivers by telephone on three random days (two weekdays, one weekend day) to conduct 24-h dietary recalls for caregiver and child intake using the standardized multiple-pass method developed for national dietary surveillance [32]. Dietary recalls were analyzed using the Nutrition Data System for Research (NDSR; Nutrition Coordinating Center, University of Minnesota). The present study focused on child consumption of fruits and vegetables, sugary drinks (e.g., soda, lemonade, fruit drinks), and fast food (e.g., McDonald’s, Pizza Hut, Crown Fried Chicken).

Per the Centers for Disease Control and Prevention (CDC) and the United States Department of Agriculture (USDA) [33], daily recommended fruit and vegetable servings for children differ by age: children 3 to 5 years are recommended to consume ≥ 2 cups while children 6 to 8 years are recommended to consume ≥ 3 cups. The 3-day average consumption of fruits and vegetables was dichotomized based on meeting or not meeting CDC/USDA recommendations. Similarly, the 3-day average sugary drink consumption was dichotomized: ≥ 1 vs < 1 12 oz serving. Finally, fast food consumption was assessed using the following question, posed during a 24-h dietary recall: *In the past seven days, how many times did your child eat from a fast food restaurant, such as McDonalds, Pizza Hut or Crown Fried Chicken?* Consistent with prior research, responses were dichotomized: ≥ 2 vs. < 2 times per week [7, 34].

*Physical activity.* Caregivers and children were instructed to wear an Actigraph GT3X (Manufacturing Technology, Inc.) accelerometer on the hip during waking hours, except when bathing or swimming, for 7 days. The Actigraph has an internal electromechanical device that detects uniaxial accelerations in the vertical plane over pre-specified time periods (i.e., epochs). A 15 s epoch was used to define children’s moderate to vigorous physical activity (MVPA), which refers to intensity of physical activity associated with health and fitness outcomes [35]. Child MVPA by age: 3 to 5 years (< 180 min/day vs. ≥ 180 min/day); 6 to 8 years (< 60 min/day vs. ≥ 60 min/day) was calculated from 7-day accelerometer data [36].

### Analytic Strategy

Descriptive sociodemographic characteristics were summarized as mean and standard deviation (SD) for continuous variables, and frequency and percentages (%) for categorical variables. Bivariate and multivariate analyses were conducted to investigate the relationships among caregiver stress types (i.e., general, parenting, race- related, and combined), caregiver coping styles (i.e., general and religious), child weight status (i.e., age and sex adjusted BMI ≥ 85th percentile), and child health behaviors (i.e., fruit and vegetable intake, sugary drink consumption, fast food consumption, and physical activity).

First, Spearman correlation coefficients were computed to verify the general relationship between caregiver stress and caregiver coping variables. Effect sizes were interpreted as small, typical, and large respectively for values of 0.10, 0.20, and 0.30 [37]. Next, logistic regression models were fit to explore associations between the independent variables (i.e., caregiver stress) and the dependent variables (i.e., child health outcomes). In the logistic regression models a composite stress score of 0 was considered the reference group as compared to a score of 1, 2, or 3. Select variables were included as a priori covariates in these logistic regression analyses. Caregiver specific covariates included: age (years), sex, body mass index (BMI), educational attainment (≤ high school vs ≥ college), annual household income (< $20,000, $25–70,000, > $70,000), relationship status (partnered/married vs single), and social support (having at least 1 other adult to help with shopping, cooking, and/or child transport). Child covariates included age (years) and sex.

Finally, caregiver coping styles that were found to have a significant relationship with caregiver stress were examined as potential moderators. In order to investigate moderation effects of caregiver coping styles on the relationship between caregiver stress and child health outcomes, interaction terms (e.g., general stress X emotional support coping) were added to the logistic regression models (see Fig. 1) [38]. Statistical significance was determined at α = 0.05. Analyses were conducted utilizing STATA 15.0 (StataCorp, College Station, TX).

## Results

**Fig. 2:**
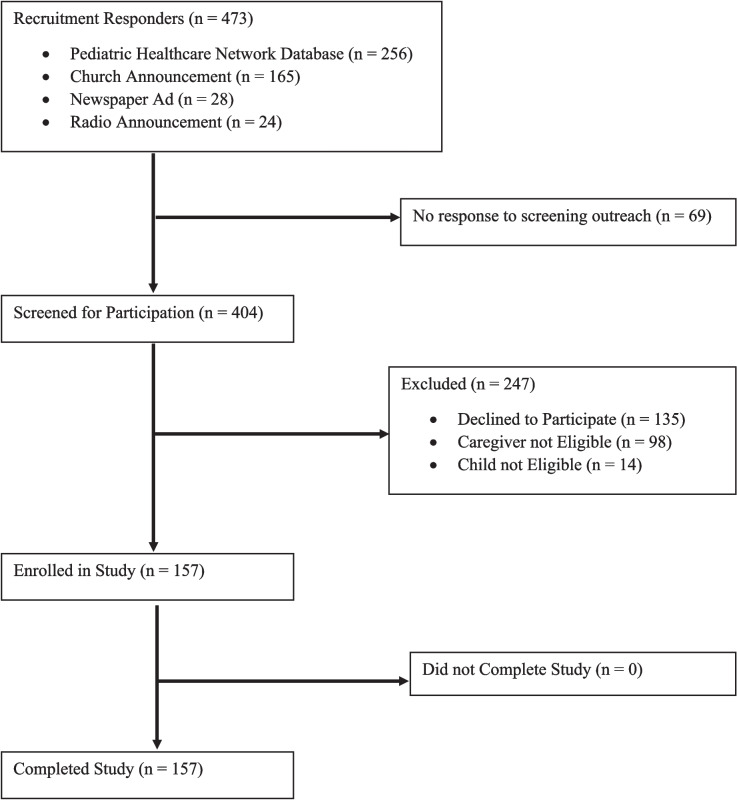
Flow Diagram

A total of 473 caregiver-child dyads responded to recruitment efforts. As seen in Fig. 2, the majority of participants were recruited from a pediatric healthcare network database and Protestant Christian churches. Most church-based participants (83%) came from a single large congregation of approximately 15,000 members. After screening, 211 caregiver-child dyads were eligible to participate; a total of 157 dyads completed the study.

As shown in Table 1, most caregivers were mothers or grandmothers (98%), and of them 81% had overweight or obesity. Although only 40% were partnered, 54% of caregivers reported having another adult who could assist with childcare responsibilities such as cooking, shopping, or transportation. This was a highly educated sample, with 80% having obtained at least some college education. In terms of children, the sample comprised 60% females; 22% of children in this study had overweight or obesity (Table 1). Although more than half (57%) of the children met recommended physical activity guidelines for age, only 27% met fruit and vegetable intake guidelines and 37% exceeded the recommended intake of fast food and sugary drinks.

**Table 1:** Descriptive Characteristics of Sample (*N* = 157)

	Frequency or (Mean)	Percentage or (SD)
**Child Variables**		
Females	94	59.9%
Age Category		
Preschool (3–5 years)	78	49.7%
School (6–7 years)	79	50.3%
BMI z-score	(0.47)	(1.04)
BMI < 85th percentile	123	78.3%
BMI ≥ 85th and < 95th percentile	34	21.7%
Met fruit and vegetable intake guidelines	42	26.8%
Consumed < 1 serving sugary drink	99	63.1%
Consumed fast food < 2 times per week	99	63.1%
Met physical activity guidelines	90	57.3%
**Caregiver Variables**		
Females	154	98.1%
Relationship to Child		
Parent	144	91.7%
Grandparent	13	8.3%
Age (years)	(35)	(7.7)
BMI (kg/m^2^)	(31.7)	(7.7)
BMI < 25	29	18.6%
BMI 25—< 30	47	30.1%
BMI ≥ 30	80	51.3%
Education Obtained		
≤ High School	31	19.7%
≥ College	126	80.3%
Annual Household Income		
< $20,000	29	19.7%
$20,000—$70,000	84	57.1%
> $70,000	34	23.1%
Partnered Relationship	62	39.5%
Adult Help	84	53.5%

*BMI* body mass index, *Adult Help* caregiver receives assistance with cooking, shopping, and/or transportation from another adult

### Bivariate Associations: Caregiver Stress and Coping

Spearman correlations revealed significant associations between coping and stress outcomes in caregivers. Specifically, the use of religion as a coping strategy (as measured by the Brief-COPE) was negatively associated with general stress (ρ = −0.16, *p* < 0.05). Using a more nuanced measure of religious coping (i.e., the Brief RCOPE), positive religious coping (i.e., spiritual strength) was negatively correlated with general stress (ρ = −0.23, *p* < 0.01) and parenting stress (ρ = −0.21, *p* < 0.01), while negative religious coping (i.e., spiritual struggle) was positively correlated with general stress (ρ = 0.26, *p* < 0.001) and parenting stress (ρ =  0.19, *p* < 0.05). Negative religious coping also showed a negative association with composite stress (ρ = −0.24, *p* < 0.01).

## Multivariate Associations: Caregiver Stress and Coping on Child Health Outcomes

Multivariate logistic regression models were fit that adjusted for caregiver and child sociodemographic factors. The first model examined associations between caregiver stress and child weight and health behavior outcomes (see Table 2) while the second model explored the moderating role of caregiver coping on the relationship between caregiver stress and child outcomes.

**Table 2:** Summary of Adjusted Logistic Regression Analysis for Caregiver Stress and Child Health Outcomes

	Child Weight Status	Child Fruit and Vegetable Intake	Child Sugary Drink Consumption	Child Fast Food Consumption	Child Physical Activity
**Caregiver Stress**	OR (95% CI)				
General Stress	1.76 (0.68 to 4.53)	0.50 (0.20 to 1.25)	2.69 (1.23 to 5.85)**	2.54 (1.17 to 5.51)*	1.10 (0.52 to 2.35)
Parenting Stress	3.37 (1.24 to 9.17)*	0.52 (0.24 to 1.17)	0.99 (0.49 to 2.039)	1.38 (0.65 to 2.93)	0.66 (0.32 to 1.33)
Race-Related Stress	1.66 (0.67 to 4.09)	0.84 (0.38 to 1.88)	1.40 (0.68 to 2.87)	1.28 (0.61 to 2.68)	1.28 (0.63 to 2.59)
Combined Stress Types	4.56 (0.96 to 21.7)	0.27 (0.06 to 1.27)	2.20 (0.68 to 7.08)	4.10 (1.19 to 15.15)*	1.33 (0.55 to 1.60)

Model adjusted for caregiver and child sociodemographic factors

^*^ = *p* < 0.05; ** = *p* < 0.01

**Fig. 3:**
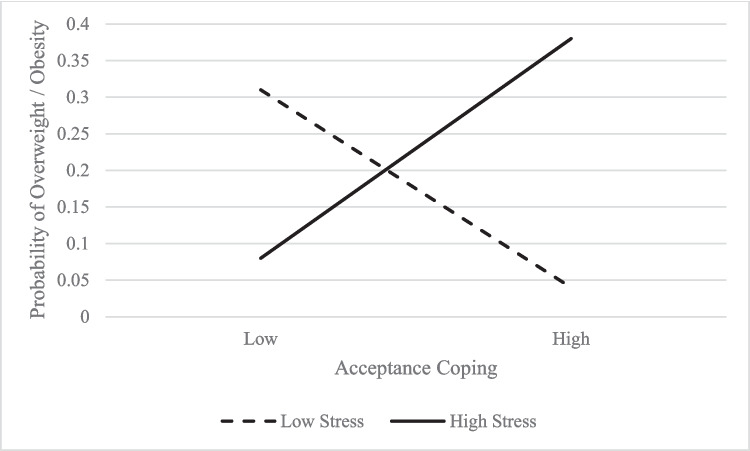
Interaction Plot: Child Weight Status on Parenting Stress X Acceptance Coping

*Child weight status.* Analyses revealed a positive association between parenting stress and child weight status such that children of caregivers with higher levels of parenting stress had three times higher odds of having overweight or obesity (OR 3.37, 95% CI 1.24 to 9.17). Exploring the interaction of caregiver coping and parenting stress revealed that in the presence of higher parenting stress and increased use of acceptance coping, children had a higher likelihood of overweight or obesity (*p* < 0.05; see Fig. 3).

**Fig. 4:**
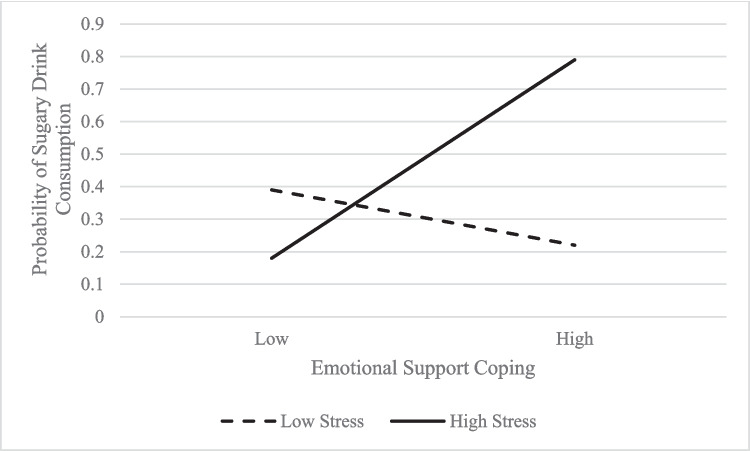
Interaction Plot: Child Sugary Drink Consumption on General Stress X Emotional Support Coping

*Child sugary drink consumption.* General stress in caregivers was associated with children consuming more than 12 oz of sugary drinks per day (OR 2.69, 95% CI 1.23 to 5.85). The interaction of emotional coping and general stress showed that when caregivers with higher levels of general stress employed emotional support coping, children consumed more sugary drinks (*p* < 0.01; see Fig. 4).

*Child fast food consumption.* The first model showed that higher general stress in caregivers is associated with 2.54 odds (95% CI 1.17 to 5.51) of a child consuming fast food two or more times per week. Analyses further revealed that children of caregivers who reported a composite of general, parenting, and race-related stress had 4 times higher odds (OR 4.1, 95% CI 1.19 to 15.15) of consuming fast food two or more times a week compared to caregivers who reported no stress types. There were no significant interactions of caregiver coping on the association of caregiver stress and child fast food consumption. Moreover, there was no association with caregiver stress and child consumption of fruits and vegetables or meeting age specific physical activity guidelines.

## Discussion

Disproportionately high rates of overweight and obesity among non-Hispanic Black youth signal a need for nuanced prevention and intervention efforts to address the elevated risks of chronic disease onset [1, 2]. Given the known linkages between caregiver experience of stress and child weight status [8], the present study sought to explore the role of caregiver stress on child weight-related health behaviors among a non-Hispanic Black sample. The study extends upon previous research by examining race-related stress as well as the accumulation of multiple stress types.

Study findings revealed that caregivers reporting higher levels of parenting stress were three times more likely to have a child with overweight or obesity. Similarly, children of caregivers endorsing higher levels of general stress were more likely to exceed recommended intake of fast food and sugary drinks. Extant literature suggests that stress associated with parenting responsibilities can influence meal preparation, food and beverage choice, supervision of free play, and transportation to structured physical activity [39, 40]. In fact, a quantitative study of non-Hispanic Black caregivers recruited from a church setting reported that when under stress, participants were more likely to eat for convenience as well as in response to their own specific food cravings, which lead to increased fast food and sugary drink consumption for their children [15]. As these health behaviors have important implications for child weight status, caregivers, and their children alike, may benefit from exposure to adaptive stress management techniques.

This study is among the first to explore the moderating effect of caregiver coping strategies on caregiver stress and child weight-related health outcomes in a minoritized sample. Children of caregivers reporting use of acceptance coping strategies (i.e., acknowledgement of and adaptation to stressors as opposed to denial or avoidance of stressors) in a context of higher parenting stress were more likely to have overweight or obesity whereas children whose caregivers employed acceptance coping while experiencing lower levels of parenting stress had decreased likelihood of excess weight. These results suggest that when caregivers are more accepting of the distress and difficulties associated with parenting, this acceptance may also extend to behaviors that influence childhood overweight and obesity. Although unconditional acceptance of a child’s weight status is considered an adaptive stance for caregivers [41], current clinical practice guidelines for childhood obesity encourage active familial support in making healthy behavior changes [1]. It must be noted that baseline parenting stress may be exacerbated by seeking to help a child meet dietary and exercise guidelines [17], which underscores the urgent need for responsive interventions to support caregivers in parenting youth with excess weight.

A similar pattern was found for caregivers who employed emotional support coping to manage higher levels of generalized stress; children of these caregivers were more likely to consume in excess of recommended sugary drink intake. Both acceptance and emotional support coping are facets of emotion-focused coping, in which an individual aims to regulate emotions associated with a difficult or stressful situation [30]. While it is well established that high levels of stress often elicit emotional responses, it will be important to further examine the role of problem-focused coping strategies in addressing child health behaviors associated with obesity – even in the face of high stress.

While positive religious coping was associated with lower levels of both general and parenting stress, religious coping was not found to influence the relationship between stress and childhood overweight and obesity or child weight-related health behaviors. Although a recent Pew Survey found Black women were more likely to say that religion is an important part of their lives [21], caregivers may not make a direct connection between their spiritual or religious beliefs and practices and their children’s health behaviors.

Interestingly, race-related distress was not directly related to child weight or associated health behaviors, though in combination with other stress types (i.e., general stress and parenting stress), was linked to higher likelihood of excess fast food consumption. Structural racism is a core social determinant of health that has been linked to numerous deleterious health outcomes, including obesity and obesity-related behaviors [18]. Individuals may experience race-related stress in response to overt or covert beliefs, policies, or practices that drive economic disadvantage or limited opportunity to engage in health behaviors. Of note, the highly educated sample represented in this present study may have been buffered against such effects. Nevertheless, current findings suggest that the accumulation of race-based stress with general and parenting stressors may play a role in obesity-related health behaviors in youth and is an important phenomenon for future study. It will also be important to explore other individual-, family-, and community-level factors that may interact with race-related distress to influence child obesity-related health outcomes.

### Strengths and Limitations

The present study has several strengths. Foremost, this study of socioeconomically diverse non-Hispanic Black dyads is unique in the exploration of moderating effects of coping styles on individual and combined stress types, including perceived racism, on childhood obesity, fast food and sugary drink consumption, and meeting guidelines for fruit and vegetable consumption and physical activity. Additionally, heights, weights, and physical activity were measured objectively. Although dietary recalls were self-reported, these data were collected at random by trained research dietitians using a rigorous protocol. It is also important to note that there was no attrition for participants who enrolled in the study; research personnel were diligent and persistent in their efforts to engage and collect data from this sample. Finally, this is the first study, to our knowledge, to control for the presence of an adult helper who assists with cooking, shopping, and transporting children to physical activity, which can be present or absent in both single-parent and partnered households.

These aforementioned strengths must be considered in light of limitations. The representation in this study of non-Hispanic Black caregivers who identify as Christian Protestants may limit generalizability of findings to caregivers and families of different racial/ethnic and faith backgrounds. Relatedly, participants were recruited from the metropolitan Philadelphia area, which limits generalizability beyond this geographic and sociocultural region. Likewise, the number of participants meeting obesity-related health guidelines varied by health behavior, which raises concern for sampling issues which may limit statistical power across analyses.

In terms of study design, the present study is a cross-sectional design and causal inferences cannot be drawn from the results. Additionally, several variables of interest were obtained from a single source: self-report questionnaires completed by caregivers, which may offer biased views of how caregivers experience stress, engage in coping strategies, and interact with their children around health behaviors; this raises concerns of common method variance. Finally, these data were collected prior to the COVID-19 pandemic, which coincided with a racial reckoning in the United States [42]. These events affected and continue to affect multiple aspects of health and functioning examined in this study, including access to nutritious foods and safe spaces for physical activity. More contemporary studies of stress and coping may yield different results than what was reported by the present sample.

## Conclusions

The present study extends a scant literature on how caregiver coping styles may influence the relationship between caregiver stress and child weight status, including weight-related health behaviors. While parental caregivers are in a strong position to model and support healthy behaviors for their children, the perception and experience of stress can disrupt a healthy balance. Although it seems intuitive that clinicians should facilitate caregiver coping to counteract the harms of stress, the present study indicates need for a much more nuanced approach. In addition to acknowledging the roles of sociodemographic factors such as race/ethnicity and socioeconomic status, it will be important that future research examines specific coping strategies to address varying levels and types of stress.

## Data Availability

The data that support the findings of this study are available from the lead author, EPP, upon reasonable request.
